# Some Statistical Results of the Treatment of Chest Wounds

**Published:** 1918

**Authors:** 


### *Some Statistical Results of the Treatment of Chest Wounds.*

By T. R. Elliott, Lt.-Colonel, R. A. M. C. Abs. from the *Lancet*, Sept. 8, 1917.

Whatever the mortality of chest cases may be, deaths almost alway occur in the first month. If the patient survives this date, the injury is not likely to prove either fatal or disabling.

Lung wounds in the war are accompanied by laceration of greater or less degree, and yet the after-history of hemothorax cases in general does not indicate that lung laceration delays convalescence.

A simple traumatic pneumothorax is a very benign condition, and is not likely to be followed by sepsis or serious effusion. There are no crippling restrictions to respiratory movement, such as persist with a lung that has collapsed beneath clotting blood.

In cases of aspirated sterile hemothorax, the aspiration was generally performed on some day from the 6th to the 12th after the wound, and oxygen replacement was used so as to enable the fluid to be removed as completely as possible at a single operation. A second operation was very rarely needed in France, and it never produced more than 10 oz. of fluid in any case.

Aspiration never caused any trouble or evidence of recurrent hemorrhage, and not a single one of the cases so treated developed infection later in England.

The author remarks, however, that a large hemothorax occasions a longer delay in returning to duty than small effusions. He is inclined to think that sepsis in the hemothorax may hasten recovery from the crippling effects of a large hemothorax because the digestive processes of infection tend to discharge with great rapidity the fibrinous masses of blood clot that otherwise might resist the expansion of the lung in its lower half.

He thinks it unnecessary to aspirate small effusions of about 30 oz., which extend only half way to the axilla and do not cause cardiac displacement. These are quickly absorbed by natural means. Aspiration of large effusions, however, has given good results.

In cases of septic hemothorax, the original volume of blood varied greatly. In some there was probably only an infected pleural effusion. When danger to life has been averted by a proper drainage, then all infections quickly assume a common place character. Much depends upon the proper placing of the original drainage and the satisfactory removal of carious ribs.

The author remarks, however, that the opinion has been gaining ground that active surgery undertaken as early as possible after the injury is the best means of dealing with chest wounds and of avoiding the difficulties that almost always result from retained missiles and shell fragments.

Such operative treatment should include:

Closure during the first 24 hours of all open wounds with air suction and free bleeding from within. (Drainage at an early date is to be avoided.)

The removal of all shell fragments except those of small size.

At a later date, the removal from the thorax of all other retained foreign bodies, when the danger of infection has passed away.

Mortality from chest wounds is due to hemorrhage, infection, or complicating wounds of the abdomen or spine. There is almost no late mortality in England and only about 5 o/o on the lines of communication in France. In the area of the armies, however, it amounts to 10 or 15 o/o in the medical units at an early date, and to about 10 o/o in the casualty clearing stations as a result of sepsis.

In cases developing sepsis within the chest, the mortality is very high, amounting to about 50 o/o under the present method of treatment, which is rib resection and drainage.

In cases with air suction and hemorrhage, treated only with occluding pads, mortality is exceedingly high.

Tangential wounds with severely comminuted ribs and mangling of the lung near the wound, are liable to severe streptoccic infection unless the wound tract is thoroughly cleansed.

In the majority of those with large retained fragments of shell, death follows soon after hemorrhage.

In cases with small retained projectiles, the presence of the projectile appears to bear no important relation to the septic hemothorax that occurs at the base hospitals.

The old surgical theory of non-intervention except by late drainage is justified only in those cases which do not develop sepsis (about 75 o/o). The high mortality in cases of sepsis, however, indicates the need of a wider practice of the new prophylactic method of early operative cleansing.

